# Occasional Lectures on the Practice of Medicine

**Published:** 1900-12

**Authors:** 


**Occasional Lectures on the Practice of Medicine.** By W. B. Cheadle, M.D. Pp. xiv., 324. London: Smith, Elder, & Co. 1900.

In addition to Dr. Cheadle's well-known Harveian lectures on the Rheumatism of Childhood, this work contains chapters devoted to the following subjects: The use and abuse of tonics, the clinical uses of opium, the diagnosis and treatment of certain minor disorders of childhood, constipation, rickets, and some practical points on the treatment of diseases of the lungs and air passages. To the practitioner these lectures will be invaluable, for they treat of conditions which are seldom seen in the course of his hospital training, but which are of every-day occurrence in private practice. The advice given with regard to treatment is always practical and concise, and we may instance more especially the saline treatment of constipation, the administration of dilute iron solutions, and the use of opium in infantile bowel disorders. With sound useful advice Dr. Cheadle combines a charm of literary style which is too seldom met with in medical works at the present day.

